# Anti-Tumor Effect and Neurotoxicity of Ethanol Adjuvant Therapy after Surgery of a Soft Tissue Sarcoma

**DOI:** 10.3390/curroncol30060399

**Published:** 2023-05-24

**Authors:** Yoshitaka Ban, Manabu Hoshi, Naoto Oebisu, Kumi Orita, Tadashi Iwai, Hana Yao, Hiroaki Nakamura

**Affiliations:** 1 Department of Orthopedic Surgery, Osaka City University Graduate School of Medicine, 1-4-3 Asahi-Machi, Abeno-ku, Osaka 545-8585, Japan; 2 Department of Orthopedic Surgery, Osaka Metropolitan University Graduate School of Medicine, 1-4-3 Asahi-Machi, Abeno-ku, Osaka 545-8585, Japan

**Keywords:** sarcoma, surgery, surgical margin, ethanol adjuvant therapy, anti-tumor effect, neurotoxicity

## Abstract

Wide resection is the main treatment for sarcomas; however, when they are located near major nerves, their sacrifices might affect limb function. The efficacy of ethanol adjuvant therapy for sarcomas has not been established. In this study, the anti-tumor effect of ethanol, as well as its neurotoxicity, were assessed. In vitro anti-tumor effect of ethanol as evaluated using MTT, wound healing, and invasion assays on a synovial sarcoma cell line (HS-SY-II). In vivo, an assessment was conducted in nude mice (implanted with subcutaneous HS-SY-II) treated with different ethanol concentrations after surgery with a close margin. Sciatic nerve neurotoxicity was assessed with electrophysiological and histological examination. In vitro, ethanol concentrations at 30% and higher showed cytotoxic effects in MTT assay and markedly reduced migration and invasive ability of HS-SY-II. In vivo, both 30% and 99.5% ethanol concentrations, compared to 0% concentration, significantly reduced the local recurrence. However, in the group treated with 99.5% ethanol, nerve conduction tests showed prolonged latency and decreased amplitude, and morphological changes suggestive of nerve degeneration were observed in the sciatic nerve, while the 30% ethanol did not cause neurological damage. In conclusion, 30% is the optimal concentration for ethanol adjuvant therapy after close-margin surgery for sarcoma.

## 1. Introduction

In surgery for soft tissue sarcomas (STSs) of the extremities, the main treatment strategy is to achieve an adequate wide margin and remove a part of the surrounding healthy tissue in addition to the malignant tumor to avoid local recurrence [1]. However, when major neurovascular tissues are located close to the tumors, sacrifice with these tissues leads to severe functional impairment of the affected limbs. In this regard, ensuring complete cure and preservation of limb function are sometimes a contrary and challenging issue in orthopedic oncology.

Ethanol therapy has been widely applied to clinical practice and surgery in different cancer fields [2,3]. To preserve the affected limb’s function and to minimize the tumor recurrence, intraoperative evaluation of surgical margin and ethanol adjuvant therapy for the remaining tissue after close-margin surgery were introduced; these techniques are reported to have similar functional and surgical oncological outcomes to those of previous methods [4,5].

However, several disadvantages were reported for ethanol adjuvant therapy, such as post-operative neuropathy, vascular occlusion, and infection [4]. Post-operative neuropathy was reported to be the most frequent adverse effect; partial sensory loss and incomplete paralysis of motor branches deteriorate the quality of life (QOL) of cancer survivors [5]. Accumulated reliable data on the anti-tumor effects of ethanol and its toxicity are lacking in orthopedic oncology and are necessary to establish its pros and cons.

Ethanol adjuvant therapy has been empirically applied after surgery for STSs located near major neovascular tissue [4]; however, no basic supporting experimental study to evaluate its anti-tumor effect is available. This study aimed to investigate the in vitro and in vivo anti-tumor effect of ethanol adjuvant therapy, as well as its neurotoxic effect on the sciatic nerve in an animal model.

## 2. Materials and Methods

All experimental procedures were performed in accordance with Osaka City University institutional guidelines for the Care and Use of Laboratory Animals. These experiments were approved by the Osaka City University Graduate School of Medicine (approval number: 20015).

### 2.1. In Vitro Anti-Tumor Effect of Ethanol Adjuvant Therapy

#### 2.1.1. Cell Line, Cell Culture, and Reagent

HS-SY-II, a human cell line derived from synovial sarcoma, was used in this study. HS-SY-II extracted from pleural effusion with lung metastasis by Sonobe [6] was obtained from Riken Bioresource Center (RCB2231, Kyoto, Japan) and cultured in Dulbecco’s modified Eagle’s medium (DMEM; Gibco; Thermo Fisher Scientific, Inc., Bohemia, NY, USA). The culture was supplemented with 10% fetal bovine serum (FBS; Gibco; Thermo Fisher Scientific, Inc., Bohemia, NY, USA) and 1% penicillin–streptomycin (Wako, Osaka, Japan) at 37 °C in a humidified atmosphere of 5% CO_2_. Ethanol was purchased from Wako (Lot.TPR5731).

#### 2.1.2. In Vitro Measurement of Cell Viability

In vitro, measurement of cell viability was determined using the 3-(4,5-dimethylthiazol-2-yl)-2,5-diphenyl-2H-tetrazolium bromide (MTT) cell Proliferation/Viability Assay Kit (R&D Systems) according to the manufacturer’s specifications. HS-SY-II was seeded in a 10 cm plate and had grown to approximately 100% confluence. Next, HS-SY-II was digested with 0.25% trypsin and 0.02% EDTA and centrifuged at 1200 rpm for 3 min and was plated at a density of 6.4 × 10^4^ cells per well in 96-well plates. The cells were cultured for 24 h before treatment and then exposed to ethanol for 5 min, washed once with phosphate-buffered saline (PBS). The MTT assay was conducted with five concentrations of ethanol (i.e., 0%, 10%, 20%, 30%, and 99.5%). After exposure to ethanol, an MTT assay was performed. Absorbance at 570 nm was determined using a microplate reader (Varioskan LUX; Thermo Fisher Scientific, Inc.).

#### 2.1.3. Wound Healing Assay

HS-SY-II had grown to confluence in 6-well plates and then were exposed to ethanol for 5 min and washed twice with PBS. The wound healing assay was conducted with three concentrations of ethanol (i.e., 0%, 30%, and 99.5%). The cell monolayer was wounded by scratching with a sterile 200 uL pipette tip, and the medium was exchanged. Photographs were taken at time points 0, 24, and 48 h at ×10 magnification using a Nikon ECLIPSE Ts2 microscope. The locations of the wound at each time point were fixed, and the percentage of wound healing was qualified with the use of ImageJ (National Institutes of Health) [7,8].

#### 2.1.4. Invasion Assay

The invasion assay was conducted using a 24-well BD BioCoat invasion chamber (8-um pore size, 6.4 mm diameter). Accordingly, 200 uL (FBS-free medium) of cell suspension (2.8 × 10^4^ cells) was inserted in the upper chamber, and 500 uL of DMEM with 20% FBS in the lower chamber as a chemoattachment. After incubation of cells at 37 °C in a humidified atmosphere of 5% CO_2_ for 24 h, HS-SY-II was exposed to ethanol for 5 min, washed once with PBS, and cultured in a FBS-free medium. The invasion assay was conducted with three concentrations of ethanol (i.e., 0%, 30%, and 99.5%).

After 48 h of incubation, filters were washed with PBS and then fixed with 700 uL of 4% paraformaldehyde at 4 °C for 20 min. Cells were then stained with 0.1% crystal violet for 15 min. The invasive cells were stained purple with a crystal violet stain. The cells on the upper chamber were completely removed with a cotton swab. The cells that had migrated were counted on a glass slide in five random fields using a Nikon ECLIPSE Ts2 microscope [7,8,9].

### 2.2. In Vivo Anti-Tumor Effect of Ethanol Adjuvant Therapy

For the HS-SY-II xenograft model, four-week-old female BALB/c nu/nu mice (Japan SLC, Inc., Shizuoka, Japan) were housed in a temperature-controlled, pathogen-free environment.

**Figure 1:**
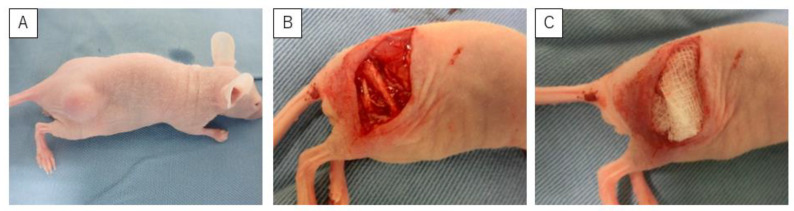
(**A**) Tumors were implanted subcutaneously in mice, and surgery was performed when the tumor exceeded 1 cm in length. (**B**) The sciatic nerve was identified after tumor resection. (**C**) A gauze soaked in ethanol was placed to cover the tissue around the sciatic nerve and over the entire surgical field.

In this stage, 45 animals were inoculated subcutaneously with HS-SY-II (1.0 × 10^6^ cells per mouse). After the subcutaneous tumors developed (>10 mm in diameter; Figure 1A), mice were randomly divided into three groups (15 mice in each group) based on the ethanol concentration: 0% ethanol (control group), 30% (30E group), and 99.5% (99.5E group). Prior to the removal of the tumor, the mice were anesthetized by subcutaneous injections of ketamine (80 mg/kg) and xylazine (10 mg/kg) into their dorsal back. A skin incision was made directly above the tumor, and the sciatic nerve was identified (Figure 1B). After surgery of the tumors with close margin, ethanol-soaked gauze was placed over the operative field, including the sciatic nerve, for 5 min (Figure 1C), based on the original suggestions for ethanol adjuvant therapy [4]. The wound was then washed with saline and closed with 5-0 nylon sutures. Recurrence-free survival (RFS) was assessed every week from week one to week eight. After eight weeks, mice were sacrificed, and sciatic nerves were harvested.

### 2.3. Neurotoxicity of Ethanol Adjuvant Therapy

#### 2.3.1. Evaluation of Electrophysiological Activity of the Sciatic Nerve

The recovery of hindlimb motor function in mice was assessed at eight weeks postoperatively based on electrophysiological recordings from the sciatic nerve as described previously [10]. Motor conduction in the sciatic nerve was assessed by stimulating the sciatic nerve.

Mice were anesthetized, and the sciatic nerve on the operated side was identified. Electrophysiological signals were acquired using a NICOLET VIKING SELECT electromyography machine (Natus Neurology Inc., Middleton, WI, USA). Nerves at the proximal end of the nerve conduit were stimulated with a unipolar 28-G needle electrode in all three groups; the recording needle electrode was positioned within the gastrocnemius muscle, while the reference needle electrode was placed at the Achilles tendon. Repetitive single pulses of 0.1 ms duration were used for a series of nerve stimulations until a maximal artifact-free compound muscle action potential (CMAP) motor response was elicited. Decreased amplitude was considered axonal degeneration, and prolonged latency as demyelinating changes.

#### 2.3.2. Histological Evaluation and Histomorphometry of the Sciatic Nerve

Electron microscopic histological analyses were performed for the sciatic nerve on the operated site in all three groups. The samples were initially fixed with a solution of 2.5% glutaraldehyde and 2% paraformaldehyde and then immersed in 1% osmium tetroxide. Subsequently, the samples were dehydrated using a series of ethanol dilutions (ranging from 50% to 100% ethanol) and impregnated with 100% epoxy resin. Semi-thin (1 μm) sections were cut and stained with toluidine blue. The myelin sheath was stained with toluidine blue.

Electron microscope histological analyses were also performed in all three groups. Ultra-thin (70 nm) sections of tissues embedded in epoxy resin were then prepared. These sections were stained with a solution of 5% uranyl acetate with 50% ethanol and Reynold’s lead citrate. The stained sections were examined using a transmission electron microscope (Talos F200C G2, Thermo Fisher Scientific, Inc., NY, USA). Twenty myelinated axons were randomly chosen from each sciatic nerve, and G-ratio values (axonal diameter/axonal and myelinated diameter) were measured using ImageJ software developed by the National Institutes of Health [11,12]. The increased G-ratio was considered to show demyelination.

### 2.4. Statistical Analysis

Data are presented as the mean ± standard deviation from at least three separate experiments. Statistical analysis was performed with a comparative test of Kruskal–Wallis followed by a Steel–Dwass post hoc analysis utilizing the Excel Statistics software package for Windows (Version 2022; Social Survey Research Information Co., Ltd., Tokyo, Japan). RFS was analyzed using the Kaplan–Meier method and compared between the three groups by log-rank test. Values of *p* < 0.05 were considered statistically significant.

## 3. Results

### 3.1. In Vitro Anti-Tumor Effect of Ethanol Adjuvant Therapy

#### 3.1.1. In Vitro Measurement of Cell Viability (MTT Assay)

**Figure 2:**
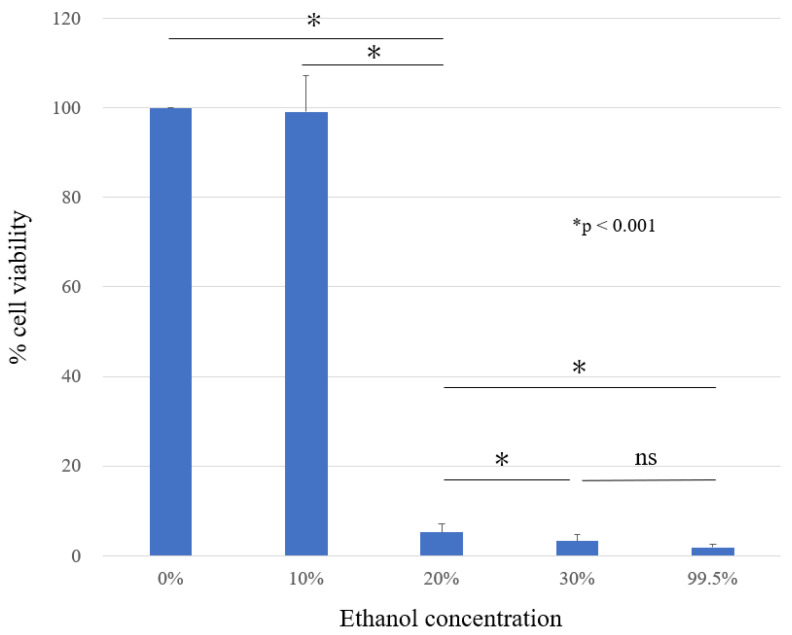
Cell proliferation determined by MTT assay in HS-SY-II. HS-SY-II were treated with different concentrations of ethanol for 5 min. The results are expressed as the % cell viability and represent the mean values ± standard deviation.* *p* < 0.001; ns: not significant.

The anti-tumor effect of ethanol on HS-SY-II was assessed by MTT assay. The cell viability of HS-SY-II in the presence of ethanol at a concentration of 0%, 10%, 20%, 30%, and 99.5% were 100 ± 0, 99 ± 8.05, 5 ± 1.98, 3 ± 1.38, and 2 ± 0.68%, respectively (Figure 2). A significantly marked decrease in cell viability was observed at 20% ethanol concentration and more compared to that in the control and 10% ethanol treatment group. A statistically significant difference was observed between the ethanol concentrations of 20% and 30% (*p* < 0.001). No significant difference was observed between ethanol concentrations of 30% and 99.5% (*p* = 0.28).

#### 3.1.2. Wound Healing Assay

**Figure 3:**
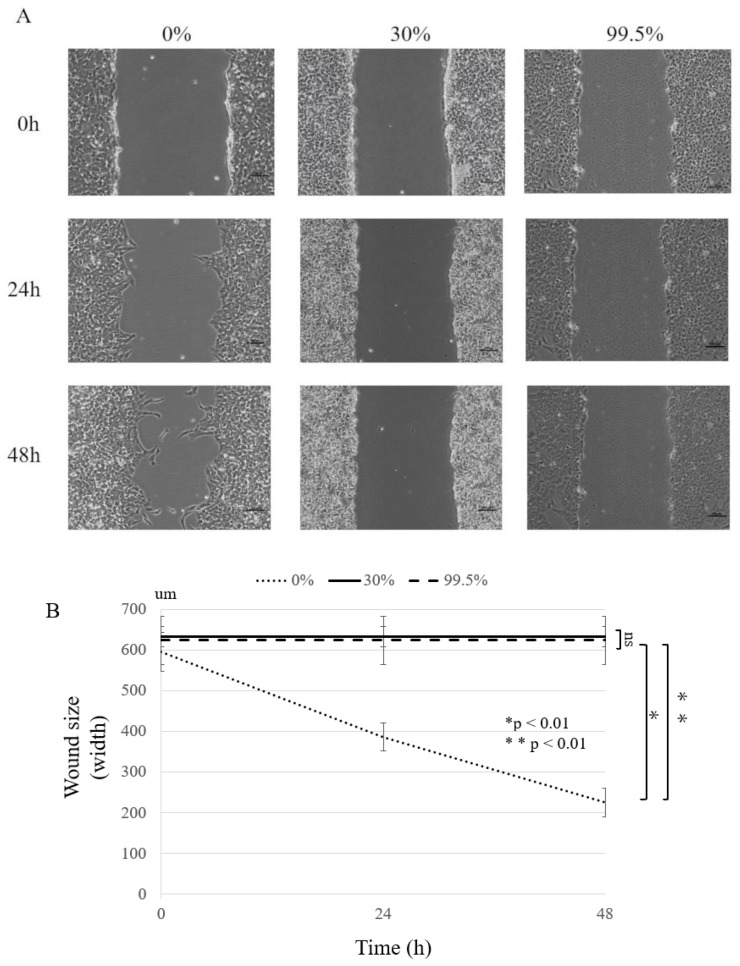
The effect of ethanol on the motility of HS-SY-II in a wound-healing assay. Recorded using a microscope at 0, 24, and 48 h after scratching the cell surface layer. (**A**) Representative images are shown for each experiment (original magnification, ×10) (**B**) The distances between wound edges of HS-SY-II at 0, 24, and 48 h. * *p* < 0.01; ** *p* < 0.01; ns: not significant.

The inhibitory effect of ethanol on the motility of HS-SY-II was assessed with a wound healing assay. In the wound healing assay, the wound size gradually decreased over 48 h at 0% ethanol concentration, but no remarkable changes in wound size were observed at 30% and 99.5% ethanol concentrations (Figure 3A). Therefore, the motility of HS-SY-II was strongly inhibited by ethanol concentrations above 30% at 48 h after scratching (*p* < 0.01; Figure 3B).

#### 3.1.3. Invasion Assay

**Figure 4:**
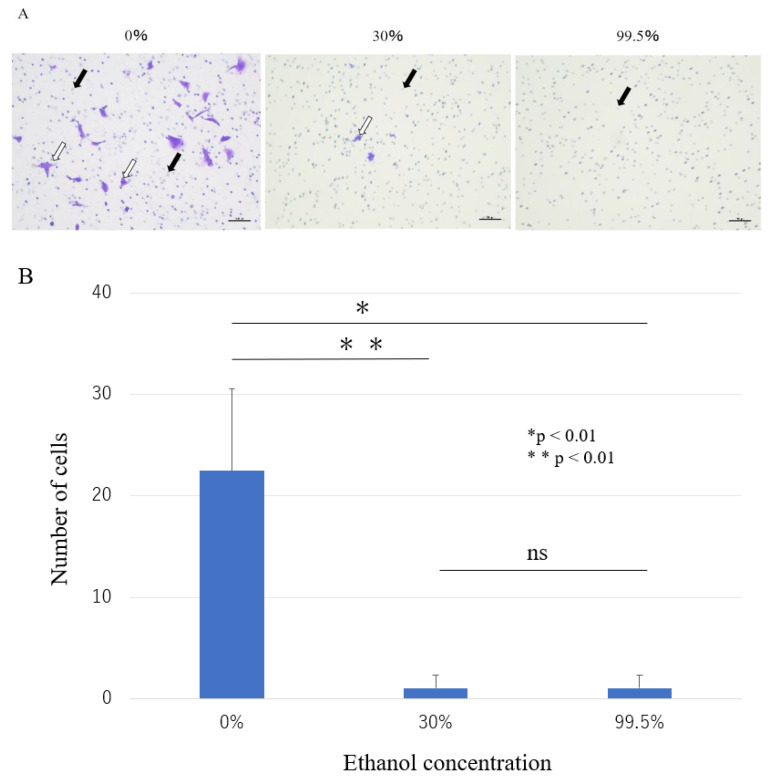
Invasion assay of HS-SY-II. HS-SY-II were treated with different concentrations of ethanol for 5 min. (**A**) The cells passed through the invasion chamber were counted after 48 h using crystal violet. (**B**) The number of cells passing through the membrane was counted in triplicate and is represented as mean ± standard deviation. White arrows indicate invasive cells, and the black arrows indicate the pore membrane.*: *p* < 0.01; **: *p* < 0.01; ns: not significant; Scale bar: 100 um.

The invasion assay was used to explore the effect of ethanol on the invasive ability of HS-SY-II. At 0% ethanol concentration, the cells passed through the invasion chamber were stained with crystal violet. At the ethanol concentrations above 30%, the number of cells passed through the invasion chamber was markedly reduced compared to that at the 0% concentration (Figure 4A). There was a statistically significant difference between the 0% concentration group and the 30% and more ethanol concentration groups. However, no significant differences were found between the 30% and the 99.5% ethanol concentration groups (*p* < 0.01; Figure 4B). Our results showed that ethanol concentrations above 30% intensely inhibit the invasive ability of HS-SY-II.

These results indicated that the in vitro anti-tumor effect of cell viability, motility, and invasion of the synovial cell sarcoma line, were observed at the 30% ethanol concentration treatment and above. Accordingly, ethanol concentrations of 0%, 30%, and 99.5% were applied to in vivo assays.

### 3.2. In Vivo Anti-Tumor Effect of Ethanol Adjuvant Therapy

**Figure 5:**
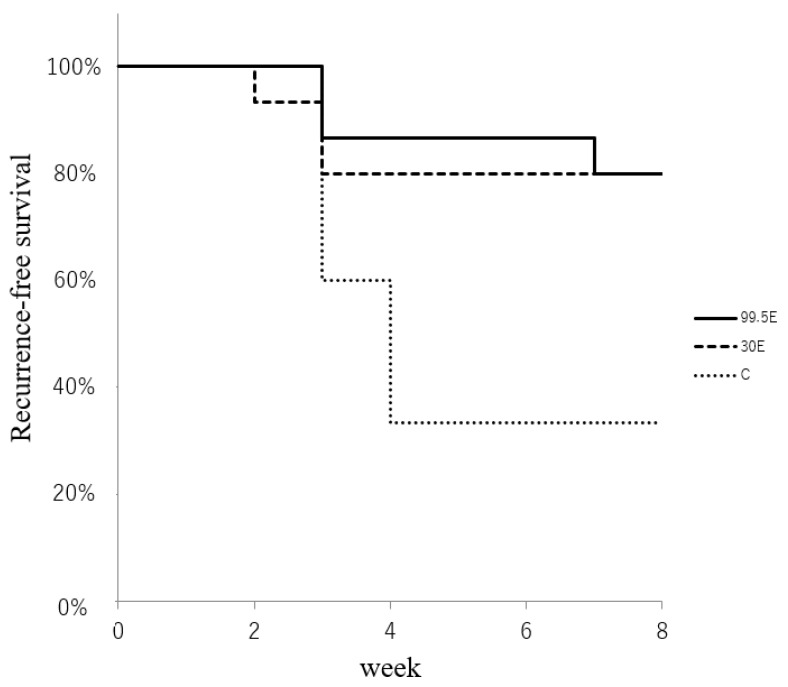
Recurrence-free survival.

Macroscopically, most mice had recurrence within four weeks after the operation, and only one mouse had recurrence after four weeks (Figure 5). The RFS at the last follow-up of eight weeks was 34%, 80%, and 80% in the control, 30E, and 99.5E groups, respectively. The log-rank test identified the statistical differences between the control and ethanol treatment groups (30E and 99.5E) but not between the 30E and 99.5E groups (*p* < 0.05; Figure 5). RFS was lower in the control group. No cases of infection occurred during this study.

### 3.3. Neurotoxicity of Ethanol Adjuvant Therapy In Vivo

#### 3.3.1. Electrophysiological Examinations of Sciatic Nerve

**Figure 6:**
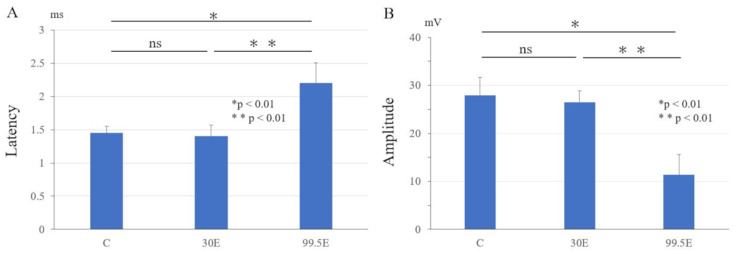
Recovery of electrophysiological results at eight weeks after the operation. Latency (**A**) and amplitude (**B**) of the compound motor action potential of the gastrocnemius. *: *p* < 0.01; **: *p* < 0.01; ns: not significant. ms: milli second; mV: milli Volt.

The latency of the gastrocnemius muscles’ CMAP in the 99.5E group (2.2 ± 0.29 ms) was the highest among all the three groups (*p* < 0.01). There was no significant difference in latency between the control group (1.45 ± 0.11 ms) and the 30E group (1.40 ± 0.08 ms; *p* = 0.98; Figure 6A). The amplitude of the gastrocnemius muscles’ CMAP in the 99.5E group (11.35 ± 4.22 mV) was the lowest among all the three groups (*p* < 0.01). There was no significant difference in amplitude between the control group (27.95 ± 3.75 mV) and the 30E group (26.5 ± 2.43 mV; *p* = 0.98; Figure 6B). These results indicated that ethanol concentration at 99.5% led to electrophysiological demyelinating changes and axonal degeneration to the sciatic nerve, but ethanol concentration at 30% treatment did not.

#### 3.3.2. Histological Evaluation and Histomorphometry for Sciatic Nerve

**Figure 7:**
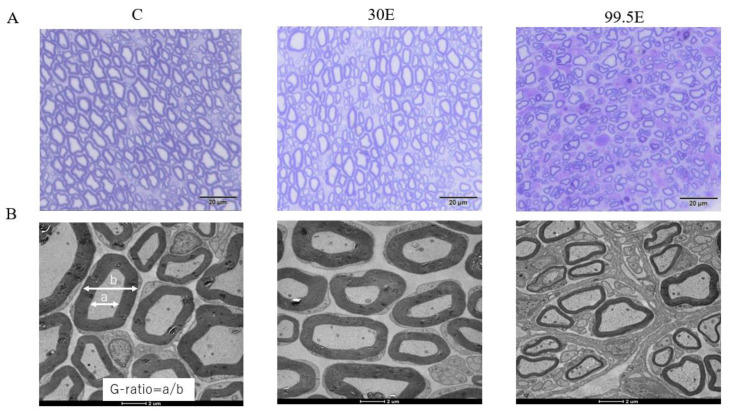
(**A**) Microscope images of cross-sectional slices of the sciatic nerve colored with toluidine blue. (**B**) Transmission electron microscope images of cross-sectional slices of the sciatic nerve. G-ratio = a/b, a: axonal diameter, b: axonal and myelinated diameter; C, control group; 30E: 30% of ethanol concentration; 99.5E: 99.5% of ethanol concentration.

Histologically, on cross-sections at the sciatic nerve, the evaluation of axons and myelin sheath of the sciatic nerve colored with toluidine blue in the control and 30E groups showed little change, whereas, in the 99.5E group, the internal structure of the nerve was clearly different from that of the normal sciatic nerve, with reduced thick myelin sheath and axon diameter compared to that in the control and 30E groups, demonstrating signs of nerve degeneration (Figure 7A).

**Figure 8:**
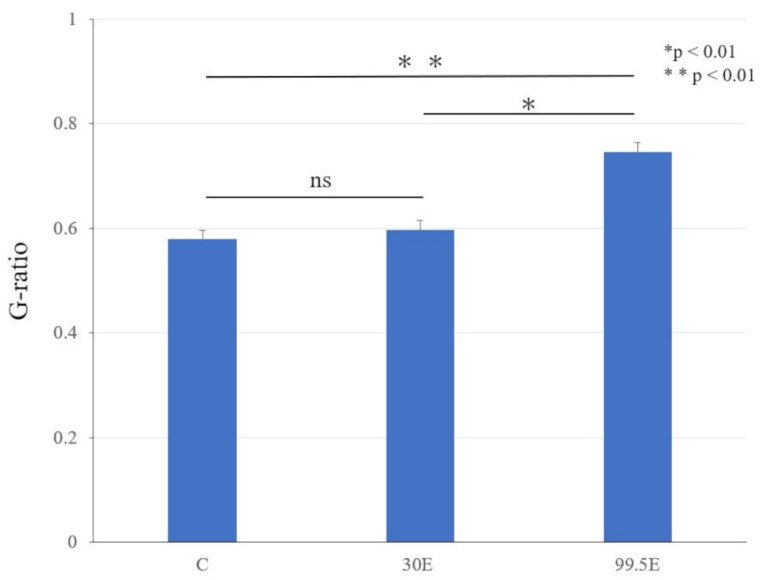
The G-ratio of the control, 30E, and 99.5E groups at eight weeks after the operation. G-ratio = a/b, a: axonal diameter, b: axonal and myelinated diameter; C, control group; 30E: 30% of ethanol concentration; 99.5E: 99.5% of ethanol concentration *: *p* < 0.01; **: *p* < 0.01; ns: not significant.

Transmission electron microscope of morphological changes revealed little morphological difference between the control and 30E groups; however, thinning of the myelin sheath structure was observed only in the 99.5E group (Figure 7B). The mean values of the G-ratio, indicating the degree of myelination, were significantly higher in the 99.5E group (0.75 ± 0.02) compared to those in control (0.58 ± 0.02) and 30E groups (0.60 ± 0.02; *p* < 0.01). The mean values of the G-ratio did not show any significant differences between the control and 30E groups (*p* = 0.17; Figure 8). Thus, the high concentration of ethanol treatment induced a remarkable decrease in axon number densities and caused thinning of the myelin sheath in the sciatic nerve of mice, indicating that 99.5E had caused tissue damage in the sciatic nerve.

## 4. Discussion

The ethanol adjuvant therapy with 30% ethanol concentration and more demonstrated anti-tumor effects on HS-SY-II, reducing the cell viability, migration function, and invasive ability. The exposure to ethanol concentrations of 30% and above after close-margin surgery on HS-SY-II in proximity to the sciatic nerve contributed to a lower recurrence rate in vivo assay. The high concentration of 99.5% ethanol inflicted neurotoxic effects on the sciatic nerve, as it was evident from abnormal electrophysiological findings suggestive of nerve degeneration. Histological evaluation and histomorphometry displayed demyelinating changes and axonal degeneration to the sciatic nerve. However, the 30% concentrations of ethanol and less did not damage the sciatic nerve. Therefore, this study proved that ethanol adjuvant therapy with 30% concentration has anti-tumor effects after surgery for STS with a close margin to the sciatic nerve without increasing the risk of neurotoxicity.

Ethanol is often used in medical fields, costs less, and is convenient. It is medically utilized for the disinfection of skin and surgical sites [13,14]. However, the anti-tumor effects of ethanol adjuvant therapy on the STS field has not been established. Anti-tumor effects of ethanol on various types of cancers have been described, and the ethanol concentration [2,3,15,16,17,18] was likely to be a determining factor of these cancer treatments. Direct ethanol injection with high concentrations in hepatocellular and thyroid carcinoma led to a reduction of tumor relapse [2,3]. However, in surgery for sarcoma, the optimal ethanol concentration should be assessed.

Surgical tumor resection with a wide margin has been widely accepted as the main treatment strategy for STSs [19]. Achieving a wide margin, covered with the surrounding healthy tissue, might reduce the local recurrence rate and result in improving overall patient survival [20,21,22]. When STSs of the extremities are in proximity to major nerves and vessels, their sacrifices could severely induce functional impairment. In this regard, ensuring the cure of the malignant tumors and preservation of the affected limb function are always contradictory, and a balance should be maintained.

The efficacy of adjuvant ethanol therapy has sometimes been applied in the field of bone and soft tissue tumors [4,23,24]. The ethanol adjuvant therapy with high ethanol concentration for major organs (such as major nerves and arteries/veins) after insufficient surgical margin has resulted in clinically comparable surgical outcomes to those reported for previous methods [25,26,27]. In this study, the efficacy of in vivo and in vitro anti-tumor effects with high ethanol concentration could be ensured. However, neurotoxicity is the most frequent adverse effect, and it should be considered [4,5] because the conventional application of adjuvant ethanol therapy for the STS field has been empirical without any experimental support. Accordingly, the investigation of optimal ethanol concentration was required.

Synovial sarcoma (SS) is an aggressive high-grade malignant tumor of an unknown tissue origin, accounting for 5–10% of all STSs [28]. This tumor commonly affects adolescents and young adults and is classified into three types: monophasic type, biphasic type (spindle cells and glandular epithelial components), and poorly differentiated type [29,30]. SS is a high-grade STS with a high recurrence rate, and recurrence is associated with a poor prognosis [31]. SS is resistant to anti-cancer therapy [32], and the significant prognostic factor is limited to wide excision [33].

In this study, our target was the thigh which is the most frequent location of STSs, being reported to account for approximately 38% of all STSs [19]. STSs arise in proximity to the sciatic nerve [34,35]. The sciatic nerve of the thigh has been preferentially selected for in vivo animal experiments [36,37].

The results of the MTT assay showed that cytotoxicity appeared stronger when the ethanol concentration exceeded 30%, which was comparable to the 99.5% ethanol concentration. Low concentrations of ethanol increase intracellular calcium concentrations and activate caspase 3/8, which induces apoptosis, thereby inducing an anti-tumor effect in vitro [15,16]. Several reports have shown that the addition of low concentrations of ethanol to anti-cancer drugs, such as doxorubicin and hyperthermia therapy, could enhance the effects of these treatments [16,17]. On the other hand, high concentrations of ethanol are known to have a dehydration effect, strong protein coagulation and denaturation, and necroptosis [18,38]. In a previous report, 27.8% of cervical carcinoma cell lines showed cell death when the ethanol concentration was 21.25%, while 99% of cells showed cell death when the ethanol concentration was 42.5% or higher [39]. In other carcinomas, most cells have been found to be killed when exposed to 15–25% ethanol concentration for 5–10 min [40]. Therefore, different anti-tumor effects of ethanol were obtained according to three factors: type of carcinoma, ethanol concentration, and exposure time. Regarding the cytotoxicity of ethanol, especially for HS-SY-II, the effective concentration and exposure time of ethanol appear to be ≥30% and 5 min, respectively. Although migration or invasive capacity has not been evaluated with high concentrations of ethanol in the past, previous reports have reported that ethanol inhibited cell migration and invasive ability by suppressing the Notch signaling pathway (Notch1, Jagged1, and c-Myc) [41]. High concentrations of ethanol may have caused the denaturation of proteins essential to these pathways. The Notch signaling pathway is also expressed in myxoid liposarcoma and gastrointestinal stromal tumors, and similar effects may be observed in other STS [42].

The results of the in vitro assays showed that the anti-tumor effect was detected at 30% and higher ethanol concentrations. To ensure the in vivo anti-tumor effect, synovial sarcoma of HS-SY-II were implanted subcutaneously, and after tumor engraftment surgical removal with close margin, ethanol adjuvant therapy was performed with three ethanol concentrations: 0% (control group), 30%, and 99.5%. The RFS at the last observation of eight weeks was 34%, 80%, and 80% in the control, 30E, and 99.5E groups, respectively. The RFS showed comparable results at 30% and 99.5% ethanol concentrations. The rate of local recurrence was dependent on the ethanol concentration, suggesting that high concentrations of ethanol eradicated residual tumor cells in the surgical field.

Neurotoxicity is a potentially worrisome problem, and the mechanism of ethanol treatment remains unknown. An electrophysiological examination of the sciatic nerve was conducted to identify the presence of disturbed function after ethanol treatment. A high concentration of 99.5% ethanol caused prolonged latency and decreased amplitude, indicating demyelinating changes and axonal degeneration, whereas an ethanol concentration of 30% did not cause any neurological damage (Figure 6).

Histological evaluation of the sciatic nerve exposed to three ethanol concentrations (0%, 30%, and 99.5%) was performed. Toluidine blue staining demonstrated nerve degeneration in the group treated with 99.5% ethanol. The G-ratio was measured using a transmission electron microscope for myelin sheath evaluation and revealed that the width of myelin was remarkably affected after exposure to a high concentration of ethanol (i.e., 99.5%). However, less than 30% ethanol treatment did not impose morphological change on the sciatic nerves (Figure 7).

Our results proved that an ethanol concentration of 30% did not cause any neurological damage in electrophysiological function and morphology. The effects of ethanol on nerves were reported indirectly and directly, depending on the method of administration. Chronic exposure to ethanol, which is metabolically converted to acetaldehyde by alcohol dehydrogenase, P4502E1(CYP2E1), and catalase inside the body [43], is thought to cause axonal damage and demyelinating changes by decreasing myelin basic protein, neurofilament protein [44,45]. Direct exposure to high concentrations of ethanol results in non-selective destruction of neural tissue, including demyelination and Wallerian degeneration, due to denaturation of cell membrane proteins and extraction of lipid compounds [46,47], and more than 50% ethanol direct exposure has the potential to destroy the peripheral nerve [47].

There are several limitations to the present study. First, for ethanol treatment, only three concentrations of 0%, 30%, and 99.5% were applied to the in vivo experiment. Second, the number of experimental animals is small, and only one synovial sarcoma cell line was analyzed. Third, neurotoxicity was observed only for a brief period, and there is a possibility that the nerves will recover after a longer period of observation after ethanol treatment. Fourth, although electrophysiological tests and morphological evaluation are available, the evaluation of sensory nerves cannot be performed. Fifth, there is a possibility that these effects might differ between mice and humans. Sixth, in the in vivo experiments, the exposure time to ethanol was only 5 min, according to the original clinical application [4]. Prolonged exposure to ethanol might result in different effects. Seventh, only neurotoxicity was evaluated, and other adverse effects of vascular damage, skin damage, and muscle damage should be elucidated. Eighth, isopropyl alcohol has also been used extensively in the field of medicine. Further research using isopropyl alcohol will be necessary in the future. Ninth, we did not conduct experiments using ethanol concentrations of 30–99.5%. Wound healing assay and invasion assay also showed similar results. Therefore, we assumed that the anti-tumor effect was equivalent between these two ethanol concentrations. High-concentration ethanol, specifically 50% or higher, is known to induce neurotoxicity [47]. Conversely, ethanol concentrations above 30% demonstrated anti-tumor effects in the vitro experiments. The aim of this study was to determine the effective concentration of ethanol for achieving an anti-tumor effect while avoiding neurotoxicity. Therefore, in this study, experiments were conducted using three different ethanol concentrations

In conclusion, high-concentration ethanol therapy showed satisfactory in vivo and in vitro anti-tumor effects but caused electrophysiological impairment to the sciatic nerve with remarkable morphological change. However, the 30% ethanol treatment was less neurotoxic while maintaining in vivo and in vitro anti-tumor effects. Accordingly, it is suggested that a 30% concentration of ethanol adjuvant therapy after surgery for sarcoma with a close margin is optimal, considering its anti-tumor effects and neurotoxicity.

## Data Availability

The data presented in this study are available from the corresponding author upon reasonable request.
